# 874. Rapid Prediction of Antibiotic Susceptibility in Blood Stream Infections Using Direct Sequencing from Blood Cultures Coupled with Neighbour-Typing Prediction Algorithms.

**DOI:** 10.1093/ofid/ofad500.919

**Published:** 2023-11-27

**Authors:** Andrew Purssell, Amanda C Carroll, Natalia Puchacz, Leanne Mortimer, Derek MacFadden

**Affiliations:** The Ottawa Hospital Research Institute, Ottawa, Ontario, Canada; The Ottawa Hospital Research Institute, Ottawa, Ontario, Canada; The Eastern Ontario Regional Laboratory Association, Ottawa, Ontario, Canada; The Eastern Ontario Regional Laboratory Association, Ottawa, Ontario, Canada; The Ottawa Hospital Research Institute, Ottawa, Ontario, Canada

## Abstract

**Background:**

Increasing rates of antimicrobial resistance in Gram-negative (GN) bacteria make empiric treatment challenging. Culture-based techniques form the basis of pathogen and antibiotic susceptibility determination yet are limited by long turn-around-times that can prolong the time a patient remains on inappropriate therapy. Direct sequencing of positive blood cultures coupled with neighbour-typing prediction algorithms could help anticipate antibiotic susceptibility and improve selection of empiric therapy. We sought to develop a rapid metagenomic diagnostic workflow and evaluate its performance for blood stream infections.

**Methods:**

We developed a metagenomic workflow for pathogen identification and prediction of antibiotic susceptibilities outlined in Figure 1. We performed Nanopore-based metagenomic sequencing on positive blood cultures from critically ill patients admitted to a quaternary care center. Isolates belonging to six common GN blood stream pathogens were subjected to a rapid lineage-based prediction algorithm (RASE) that predicts antibiotic susceptibility against a reference set of local isolates. We calculated test performance of predictions compared to phenotypic susceptibility testing. Impact of prediction on post-test probability was calculated for each agent and in aggregate benchmarked against minimum susceptibility thresholds for empiric treatment of 80% for mild infections and 90% for moderate-severe infections.

**Figure:**
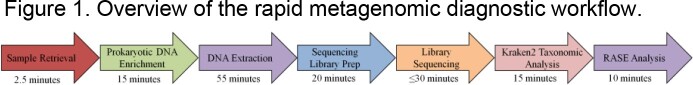

**Results:**

We performed metagenomic sequencing and RASE analysis on twelve unique GN blood culture samples with predictions available in 2.5 hours. Across all organisms and antibiotics tested, sensitivity and specificity were 0.87 (95% CI 0.76-0.94) and 0.56 (95% CI 0.31-0.78) although these values approached one for some agents. Impact of RASE predictions on probability of susceptibility is shown in Figure 2. For all antibiotics combined, baseline susceptibility was 79% but improved to 88% or reduced to 47% when RASE predicted a susceptible or resistant phenotype respectively, an effect echoed by specific agents tested.

**Figure:**
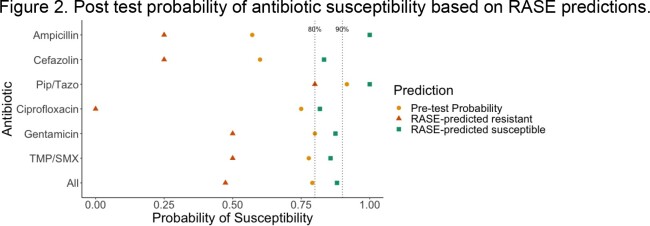

Probability of susceptibility to specific agents or in aggregate after prediction of a susceptible or resistant phenotype by RASE. This was benchmarked against minimum susceptibility thresholds for empiric treatment of 80% for mild infections and 90% for moderate-severe infections.

**Conclusion:**

We developed a novel metagenomic diagnostic workflow for rapidly predicting antibiotic susceptibility of common GN blood stream pathogens that could improve early selection of empiric therapy.

**Disclosures:**

**All Authors**: No reported disclosures

